# Selenium-Containing Protein From Selenium-Enriched *Spirulina platensis* Attenuates Cisplatin-Induced Apoptosis in MC3T3-E1 Mouse Preosteoblast by Inhibiting Mitochondrial Dysfunction and ROS-Mediated Oxidative Damage

**DOI:** 10.3389/fphys.2018.01907

**Published:** 2019-01-09

**Authors:** Jing-yi Sun, Ya-jun Hou, Xiao-yan Fu, Xiao-ting Fu, Jin-kui Ma, Ming-feng Yang, Bao-liang Sun, Cun-dong Fan, Jinrok Oh

**Affiliations:** ^1^Department of Orthopedic Surgery, Wonju Severance Christian Hospital, Wonju College of Medicine, Yonsei University, Wonju, South Korea; ^2^Key Lab of Cerebral Microcirculation in Universities of Shandong, Taishan Medical University, Taishan, China; ^3^Faculty of Bioresource Sciences, Akita Prefectural University, Akita, Japan

**Keywords:** osteoporosis, osteoblasts dysfunction, selenium-containing protein, cancer chemotherapy, mitochondrial dysfunction, oxidative damage, apoptosis

## Abstract

Accumulated evidences have verified that cancer chemotherapy may increase the risk of osteoporosis and severely affected the life quality. Osteoclasts hyperactivation was commonly accepted as the major pathogenesis of osteoporosis. However, the role of osteoblasts dysfunction in osteoporosis was little investigated. Our previous study has confirmed that selenium-containing protein from selenium-enriched *Spirulina platensis* (Se-SP) exhibited enhanced hepatoprotective potential through inhibiting oxidative damage. Herein, the protective effect of Se-SP against cisplatin-induced osteoblasts dysfunction in MC3T3-E1 mouse preosteoblast was investigated, and the underlying mechanism was evaluated. The results indicated that cisplatin dramatically decreased cell viability of preosteoblast by triggering mitochondria-mediated apoptosis pathway. Cisplatin treatment also caused mitochondrial dysfunction and reactive oxide species (ROS)-mediated oxidative damage. However, Se-SP pre-treatment effectively prevented MC3T3-E1 cells from cisplatin-induced mitochondrial dysfunction by balancing Bcl-2 family expression and regulating the opening of mitochondrial permeability transition pore (MPTP), attenuated cisplatin-induced oxidative damage through inhibiting the overproduction of ROS and superoxide anion, and eventually reversed cisplating-induced early and late apoptosis by inhibiting PARP cleavage and caspases activation. Our findings validated that Se-SP as a promising Se species could be a highly effective way in the chemoprevention and chemotherapy of oxidative damage-mediated bone diseases.

## Introduction

Osteoporosis as a metabolic disease was characterized by low bone mass, microstructure damage, fragility and proneness to fracture, which severely affects the life quality of patients (Zhang et al., 2016). Chemotherapy as one of the important ways in treating human cancers has achieved great success (Palchaudhuri and Hergenrother, 2007; Chen et al., 2010). However, the severe side effects have limited its clinic application, including liver toxicity, cardiotoxicity, nephrotoxicity, neurotoxicity, and osteocytotoxicity (Markman, 2003; Bruijnincx and Sadler, 2008). Increasing studies have confirmed that cancer patients suffering from cancer chemotherapy showed an increased risk of osteoporosis, which lead to decreased life quality and high mortality (Cooper, 1997; Hoff and Gagel, 2005). Cancer chemotherapy-induced overproduction of reactive oxygen species (ROS), and subsequent induction of oxidative damage and abnormal apoptotic cell death all contributed to anticancer drugs-induced side effects (Tuncer et al., 2010; Soliman et al., 2016). Osteoclasts hyperactivation was commonly accepted as the major pathogenesis of osteoporosis. However, little information about the role of osteoblasts dysfunction in osteoporosis was available. Therefore, investigation about oxidative damage-induced osteoblasts dysfunction has attracted much attention.

Selenium (Se) as an essential trace element for human showed multiple pharmacological properties, including antioxidant, anti-tumor activities, and enhancement of immunity (Rayman, 2000; Brown and Arthur, 2001; Schomburg, 2016). Se was metabolized in biological environment, and eventually incorporated into Se-containing proteins (Rayman, 2008; Zhang et al., 2011; Schmidt and Simonovic, 2012). Se can function in the active sites of kinds of Se-containing enzymes and displays novel protective potential against oxidative damage-mediated diseases (Brown and Arthur, 2001; Chen and Wong, 2009b). *Spirulina platensis* (*S. platensis*) is rich in proteins, vitamins and other nutritional elements, and has been emerging as the potential resource of functional food (Sayed et al., 2015). *S. platensis* was a good carrier of Se, and accumulated Se can be incorporated into the proteins of *S. platensis*. Our previous studies have confirmed that Se-containing protein from Se-enriched *S. platensis* (Se-SP) after Se incorporation have achieved enhanced antioxidant potential against oxidative damage-induced hepatotoxicity and erythrocytes injury (Zhang et al., 2011; Fan et al., 2012). However, whether Se-SP can attenuate cisplatin-induced osteoblasts dysfunction in MC3T3-E1 mouse preosteoblast has not been explored, and the underlying mechanism remains unclear.

## Materials and Methods

### Chemicals

TUNEL-DAPI kit, JC-1 pobe, DCFH-DA probe, Mito-SOX probe, PI staining solution and MTT were bought from Beyotime company (Shanghai, China). DMEM medium, fetal bovine serum (FBS), and penicillin–streptomycin were purchased from Invitrogen (Carlsbad, CA, United States). All antibodies and inhibitors used in the present study were purchased from Cell Signaling Technology (Beverly, MA, United States). Water used in this study was supplemented by Milli-Q system from Millipore.

### Culture of Se-Enriched *S. platensis*

*Spirulina platensis* was cultured in a 1000 ml flask with 600 ml Zarrouk medium (pH 9.0) at 30°C receiving a 14:10 h light:dark cycle. Culture of Se-enriched *S. platensis* was carried out by a stepwise Se addition method as previously reported (Fan et al., 2012). Briefly, Se in the form of sodium selenite (Na_2_SeO_3_) was added to the culture medium with the dosage at 100 mg/l (day-7), 150 mg/l (day-8), and 200 mg/l (day-9). The accumulated Se concentration in Zarrouk medium was set as 450 mg/l. *S. platensis* (without Se) was employed as the negative control. Morphology of *S. platensis* cells were identified by phase contrast microscope and fluorescence microscope. *S. platensis* cells and Se-enriched *S. platensis* were both harvested and freeze dried on day-11 at -20°C for further use.

### Extract of Se-Containing Protein (Se-SP)

*Spirulina platensis* protein (SP) and Se-containing *S. platensis* protein (Se-SP) were extracted as previously reported (Fan et al., 2012). Briefly, *S. platensis* cells were freeze-dried and suspended in PBS (50 mM, pH 7.0). Then, cells were given ultrasonication 3 min (Sonics VCX 600 system, 200 W), and the total protein of SP and Se-SP were prepared by centrifuge at 11,000 *g* for 30 min. SP and Se-SP were quantified by BCA kit for further use.

### Cell Culture and Cellular Uptake of Se

MC3T3-E1 mouse preosteoblast cells line was purchased from ATCC (Manassas, VA, United States), and cultured in DMEM medium supplemented with 10% FBS and 1% penicillin-streptomycin. Cells were incubated at 37°C with a 5% CO_2_ atmosphere. Cellular uptake of Se in MC3T3-E1 cells was determined by ICP-AES method (Chen and Wong, 2008). Briefly, MC3T3-E1 cells (8 × 10^5^cells) seeded in dish were exposed to 80 μg/ml Se-SP for 0–24 h, or 0–80 μg/ml Se-SP for 24 h. Cells after treatment were collected and re-suspended to 10^7^ cells/sample. Then, cells was digested with nitric acid and H_2_O_2_, and diluted to 10 ml with H_2_O and employed for total Se determination (μg/10^7^ cells).

### Cell Viability

MC3T3-E1 cells (8 × 10^3^cells/well) seeded in 96-well plate were treated with 0–80 μg/ml cisplatin for 24 h, or cells were treated with 80 μg/ml SP or Se-SP for 48 h. For protective treatment, cells were pre-incubated with 5–20 μg/ml Se-SP for 24 h, or/and co-treated with 40 and 80 μg/ml cisplatin for another 24 h. After treatment, cell morphology was observed by phase contrast microscope (magnification, 200×), and cell viability was examined by MTT method.

### Cell Apoptosis and Cell Cycle Distribution

MC3T3-E1 cells seeded in 6-well plate (10^5^cell/well) were cultured for 24 h for adherence. Cells were pre-treated with or without 10 μg/ml Se-SP for 24 h, and cells were co-treated with or without 40, 60, and 80 μg/ml cisplatin for another 24 h. Then, cells were harvested, washed with PBS, fixed by ethanol and stained with PI. Cells late apoptosis and cell cycle distribution were analyzed by flow cytometry. Sub-G1 peak was measured to reflect the cells apoptosis. Multi-Cycle software was used to analyze the cell cycle in G0/G1, S, and G2/M. 10^4^ cells/sample were recored.

### Annexin V-FITC/PI Staining

Cells early apoptosis was examined by labeling the phosphatidylserine protein in intracellular surface with an annexin V-FITC/PI staining kit. Briefly, MC3T3-E1 cells seeded in glass slides were pre-treated with 10 μg/ml Se-SP for 6 h, and co-treated with 80 μg/ml cisplatin for 6 h. Then, the living cells were incubated with annexin V-FITC and PI probes, respectively, according to the manufacturer’s instructions. Then, cells early apoptosis was examined by imaging the phosphatidylserine protein under a fluorescence microscope (Nikon Eclipse80i).

### Evaluation of Mitochondrial Membrane Potential (ΔΨm)

MC3T3-E1 cells were labeled with 10 μg/ml JC-1 probe at 37°C for 10 min. Then, cells were pre-treated with 10 μg/ml Se-SP for 6 h or/and 80 μg/ml cisplatin for 6 h. Then, cells were washed and the intracellular ΔΨm was observed using a fluorescence microscope (Nikon Eclipse80i).

### Measurement of ROS and Superoxide Anion

Intercellular ROS and superoxide anion were imaged by DCFH-DA and Mito-SOX, respectively. Briefly, cells seeded in 6-well plate (10^5^cell/well) were pre-treated with 10 μg/ml Se-SP for 24 h, and co-treated with 80 μg/ml cisplatin for 24 h. Then, cells were incubated with 10 μg/ml DCFH-DA (green) or Mito-SOX (red) at 37°C for 15 min. Then, cells were washed and observed using a fluorescence microscope (Nikon Eclipse80i).

### Western Blot Analysis

Cells seeded in 9-cm dish were pre-treated with 10 μg/ml Se-SP for 24 h, and co-treated with 80 μg/ml cisplatin for 24 h. Total protein was extracted and quantified by BCA kit. Then, 40 μg total protein was loaded for SDS–PAGE electrophoresis. After electrophoresis, the proteins in gels were transferred onto a nitrocellulose membrane. Then, the membrane was incubated with 5% non-fat milk, primary antibodies, and secondary antibodies, respectively. Then, the proteins protein was imaged under a Bio-Rad Imaging System.

### Statistical Analysis

All experiments were done three times and data were expressed as means ± SD. SPSS 13.0 was conducted to analyze the statistical analysis. The statistical analysis between two groups was analyzed by a Student’s *t*-test. Multiple comparisons was used to analyze the statistical analysis among three or more groups. “^∗^” or “^∗∗^” was used to indicate the *P <* 0.05 or *P <* 0.01, respectively. Different characters was used to indicate the significance at *P* < 0.05 level.

## Results

### Identification of Se-Enriched *S. platensis* and Cellular Uptake of Se

**FIGURE 1:**
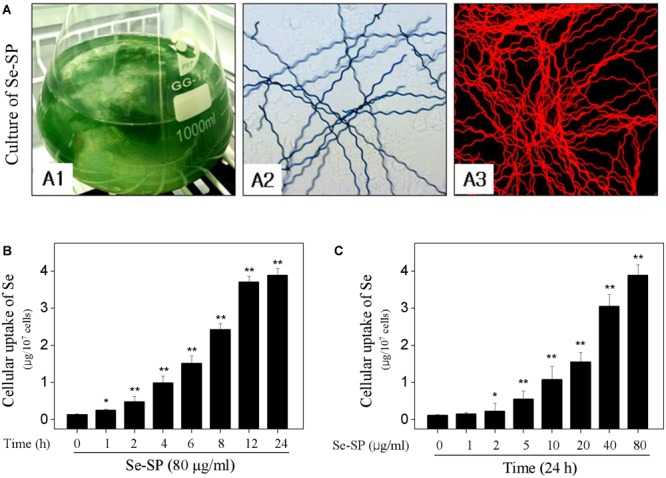
Culture of Se-enriched *Spirulina platensis* and cellular uptake of Se. **(A)** Culture of Se-enriched *S. platensis*. **(A1)**: Se-enriched *S. platensis* was cultured in 1000 ml Erlenmeyer flasks with Zarrouk medium. **(A2)**: Phase contrast of Se-enriched *S. platensis*. **(A3)**: Fluorescent morphology of Se-enriched *S. platensis*. Time-dependent **(B)** and dose-dependent **(C)** cellular uptake of Se. MC3T3-E1 cells were treated with 80 μg/ml Se-SP for 24 h, or cells were treated with 0–80 μg/ml Se-SP for 24 h. Cellular Se concentration was determined by ICP-AES method. All data and images were obtained from three independent experiments. Bars with “^∗^” or “^∗∗^” indicates *P* < 0.05 or *P* < 0.01, respectively. when compared with control group.

Selenium-enriched *S. platensis* was cultured with Zarrouk medium in a 1000 ml Erlenmeyer flask (Figure 1A1). Se-enriched *S. platensis* displayed obvious heliciform appearance (Figure 1A2), which showed no significant change in morphology compared to that of the *S. platensis*. The *S. platensis* and Se-enriched *S. platensis* both exhibited bright red fluorescence under a fluorescent microscope (Figure 1A3). Phycocyanin (PC) and allophycocyanin (APC) represent its main ingredient of *S. platensis* protein with large molecular. However, the ICP-AES result indicated that Se-SP successfully crossed the cell membrane and accumulated in MC3T3-E1 cells. As shown in Figures 1B,C, the ICP-AES results showed time-dependent and dose-dependent uptake of Se in MC3T3-E1 cells, indicating the potential application of Se-SP as a new Se form.

### Se-SP Inhibits Cisplatin-Induced Cytotoxicity in MC3T3-E1 Cells

**FIGURE 2:**
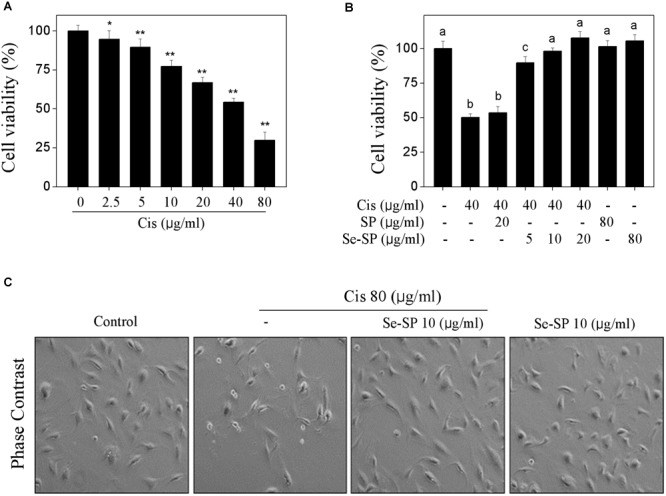
Selenium-SP inhibits cisplatin-induced cytotoxicity in MC3T3-E1 cells. **(A)** Dose-dependent cytotoxicity of cisplatin on MC3T3-E1 cells. Cells seeded in 96-well plate were treated with 0–80 μg/ml cisplatin for 24 h. **(B)** Se-SP inhibited cisplatin-induced cytotoxicity in MC3T3-E1 cells. Cells seeded in 96-well plate were pre-treated with 5–20 μg/ml SP or Se-SP for 24 h, and co-treated with 40 μg/ml cisplatin for another 24 h. Cell viability was detected by MTT assay. **(C)** Phase contrast of MC3T3-E1 cells morphology. Cells morphology was examined by phase-contrast microscopy. All data and images were obtained from three independent experiments. Bars with “^∗^” or “^∗∗^” indicates *P* < 0.05 or *P* < 0.01, respectively, when compared with control group. Bars with different characters are statistically different at *P* < 0.05 level, which achieved the multiple comparisons.

Primarily, the cytotoxicity of cisplatin toward MC3T3-E1 was tested. As shown in Figure 2A, cisplatin treatment dose-dependently inhibited MC3T3-E1 cells growth. For instance, cells exposed to 20, 40, and 80 μg/ml cisplatin for 24 h showed dose-dependent decline in cell viability from 100 to 66.8%, 54.3 and 29.8%, respectively. No significant cytotoxicity was examined after treatment of 80 μg/ml Se-SP or SP for 48 h (Figure 2B). However, Se-SP pre-treatment significantly inhibited cisplatin-induced cytotoxicity in a dose-dependent manner in MC3T3-E1 cells. As shown in Figure 2B, pre-treatment of cells with 5 and 10 μg/ml Se-SP for 24 h effectively prolonged the cell viability from 54.3 (cisplatin, 40 μg/ml) to 89.3 and 98.2%, respectively. Pre-treatment of cells with SP showed no significant protective effect (Figure 2B). Morphological improvement of MC3T3-E1 cells further confirmed Se-SP’s protective effect (Figure 2C). Taken together, addition of Se suggested that Se-SP achieved enhanced protective potential against cisplatin-induced cytotoxicity in MC3T3-E1 cells.

### Se-SP Attenuates Cisplatin-Induced Apoptosis in MC3T3-E1 Cells

**FIGURE 3:**
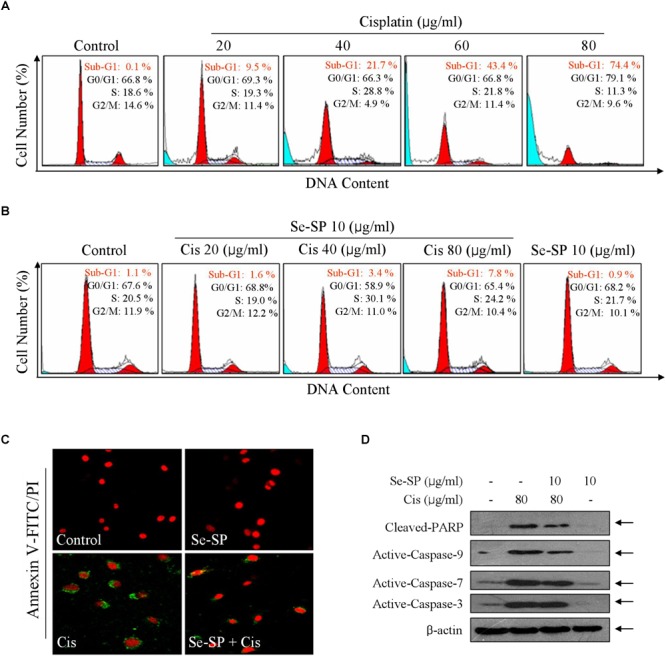
Selenium-SP reverses cisplatin-induced apoptosis in MC3T3-E1 cells. **(A)** Cisplatin-induced late apoptosis. Cells were treated with 20–80 μg/ml cisplatin for 24 h, and cells late apoptosis was examined by flow cytometric analysis. **(B)** Se-SP reversed cisplatin-induced late apoptosis in MC3T3-E1 cells. Cells were pre-treated Se-SP and co-treated with or without cisplatin. Then, Cells late apoptosis were analyzed by flow cytometric analysis. **(C)** Cells early apoptosis by annexin V-FITC/PI. Cells were pre-treated with 10 μg/ml Se-SP for 6 h, and/or co-treated with 80 μg/ml cisplatin for 6 h. Then, cells early apoptosis in living cells was imaged by annexin V-FITC/PI kit according to the manufacturer’s instructions. **(D)** Se-SP attenuated cisplatin-induced PARP cleavage and caspases activation. Cells seeded in 9-cm disk were pre-treated with 10 μg/ml Se-SP for 24 and/or co-treated with 80 μg/ml cisplatin for 24 h. Protein expression was assayed by western blotting method. All data and images were obtained from three independent experiments. Bars with different characters are statistically different at *P* < 0.05 level.

Cell death mechanism was subsequently explored by flow cytometry. As shown in Figure 3A, cisplatin treatment caused significant cells late apoptosis in MC3T3-E1 cells with a dose-dependent manner, as convinced by the increase of Sub-G1 peak. For instance, treatment of cells with 20, 40, and 80 μg/ml cisplatin induced significant cells late apoptosis from 1.1 (control) to 9.5, 21.7, and 74.4%, respectively (Figure 3A). However, Se-SP pre-treatment significantly inhibited cisplatin-induced late apoptosis to 1.6, 3.4, and 7.8%, respectively (Figure 3B). Se-SP treatment alone caused no significant cells late apoptosis. Cells early apoptosis was also detected by annexin V-FITC/PI staining. As shown in Figure 3C, MC3T3-E1 cells exposed to cisplatin showed noticeable cells early apoptosis, as indicated by the ectropion of phosphatidylserine protein convinced by the green fluorescence. As expected, Se-SP pre-treatment effectively attenuated cisplatin-induced cells early apoptosis. Moreover, the protective mechanism of Se-SP against cisplatin-induced apoptosis was further investigated in protein level by western blotting. As shown in Figure 3D, treatment of MC3T3-E1 cells with cisplatin alone induced significant PARP cleavage and activation of caspase-3, caspase-7 and caspase-9, indicating the activation of mitochondria-mediated apoptosis pathway. However, Se-SP pre-treatment markedly attenuated cisplatin-induced PARP cleavage and caspases activation. Taken, together, our findings suggested that Se-SP had the potential to attenuate cisplatin-induced cells early and late apoptosis in MC3T3-E1 cells through inhibiting PARP cleavage and caspases activation.

### Se-SP Blocks Cisplatin-Induced Mitochondrial Dysfunction in MC3T3-E1 Cells

**FIGURE 4:**
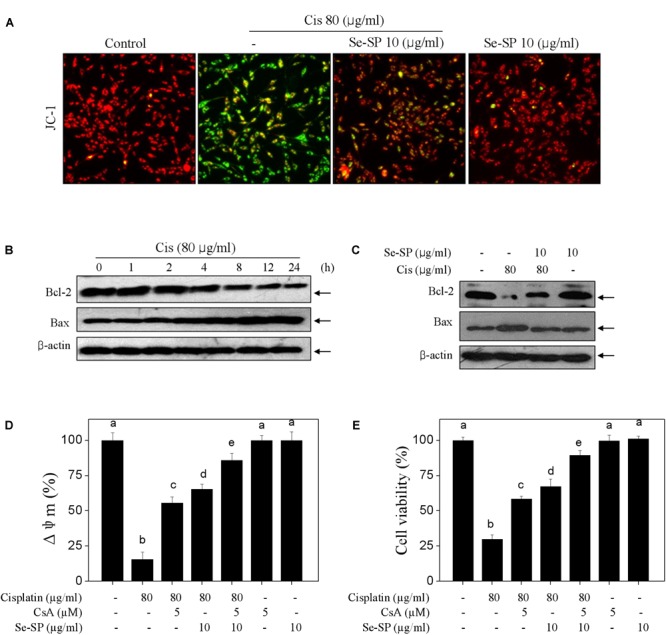
Selenium-SP blocks cisplatin-induced mitochondrial dysfunction. **(A)** Se-SP blocked cisplatin-induced loss of mitochondrial membrane potential (Δψm). MC3T3-E1 cells seeded in 6-well plate were pre-treated with 10 μg/ml Se-SP for 24 and/or co-treated with 80 μg/ml cisplatin for 24 h. Cells after treatment were labeled by JC-1 probe. The fluorescent shift from red to green was employed to indicate the loss of Δψm. **(B)** Time-dependent effect of cisplatin on Bcl-2 family. Cells were treated with 80 μg/ml cisplatin for 0–24 h. **(C)** Se-SP rescued cisplatin-induced Bcl-2 family imbalance. Bcl-2 and Bax were examined by western blotting. Effect of CsA on Δψm **(D)** and cell viability **(E)**. Cells were pre-treated with 5 μM CsA for 2 h before combined treatment. Cell viability and Δψm were detected by MTT assay and JC-1 staining, respectively. All data and images were obtained from three independent experiments. Bars with different characters are statistically different at *P* < 0.05 level.

Mitochondria-mediated apoptosis was considered to be the major mechanism in cisplatin-induced cell death (Fan et al., 2017a). Hence, mitochondrial function was fully elucidated in cisplatin-treated MC3T3-E1 cells. Firstly, the mitochondrial membrane potential (Δψ_m_) was monitored with JC-1 probes. As shown in Figure 3A, cisplatin treatment caused significant loss of Δψ_m_, as imaged by the green fluorescence (Figure 4A). Se-SP pre-treatment completely blocked cisplatin-induced the depletion of Δψ_m_ in MC3T3-E1 cells (Figure 4A). Bcl-2 family was the main factor in regulating mitochondrial permeability and inducing apoptosis. Therefore, a time-course effect of cisplatin on Bcl-2 family was examined. As show in Figure 4B, cisplatin treatment caused continuous decrease of Bcl-2 at 4 h. Bax expression in cisplatin-treated MC3T3-E1 cells showed significant increase at 4 h. However, the Bcl-2 and Bax expression in cisplatin-treated cells were effectively normalized by Se-SP pre-treatment (Figure 4C). To further emphasize the significance of Δψ_m_, cyclosporine (CsA), an inhibitor of mitochondrial permeability transition pore (MPTP) was employed. As shown in Figures 4D,E, MPTP inhibition by CsA significantly improved the Δψ_m_ and cell viability in cisplatin-treated MC3T3-E1 cells. Combined treatment of CsA and Se-SP achieved enhanced improvement of Δψ_m_ and cell viability, indicating that Se-SP can act as a natural inhibitor of MPTP to regulate mitochondria-mediated apoptosis. Taken together, the result suggested that Se-SP blocked cisplatin-induced mitochondrial dysfunction by regulating Bcl-2 family and MPTP opening.

### Se-SP Suppresses Cisplatin-Induced Oxidative Damage in MC3T3-E1 Cells

**FIGURE 5:**
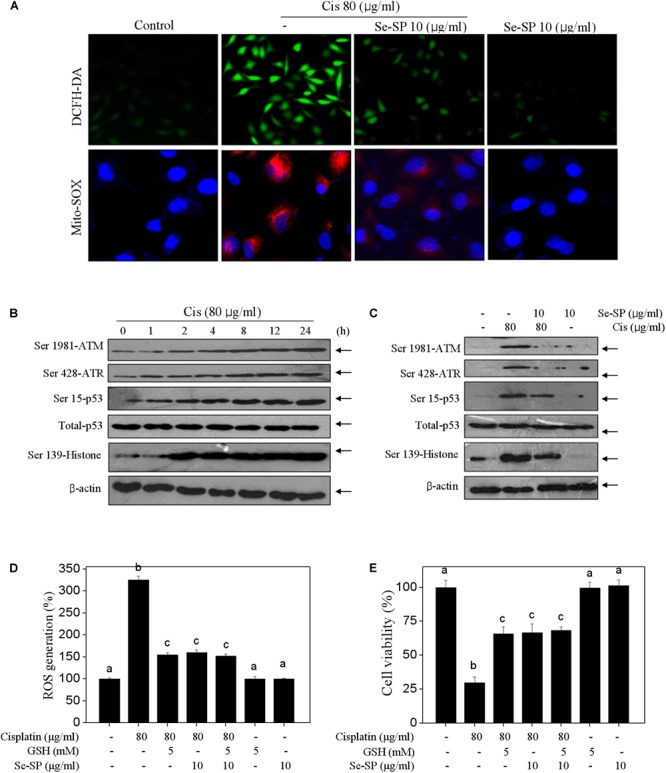
Selenium-SP attenuates ROS-mediated oxidative damage in cisplatin-treated MC3T3-E1 cells. **(A)** Se-SP prevented cisplatin-induced accumulation of ROS and superoxide production. MC3T3-E1 cells seeded in 6-well plate were pre-treated with 10 μg/ml Se-SP for 24 h and/or co-treated with 80 μg/ml cisplatin for 24 h. Cells after treatment were labeled by DCFH-DA (green) or Mito-SOX (red) for detection of ROS and superoxide production, respectively. **(B)** Time-dependent effect of cisplatin on DNA damage signal axis. Cells were treated with 80 μg/ml cisplatin for 0–24 h. **(C)** Se-SP attenuated cisplatin-induced DNA damage. Protein expression was examined by western blotting. Effect of GSH on ROS generation **(D)** and cell viability **(E)**. Cells were pre-treated with 5 mM GSH for 2 h before combined treatment. Cell viability and ROS generation were detected by MTT assay and DCFH-DA staining, respectively. All data and images were obtained from three independent experiments. Bars with different characters are statistically different at *P* < 0.05 level.

Reactive oxide species-mediated oxidative damage contributes to the main mechanism of cisplatin-based chemotherapy (Soliman et al., 2016). Therefore, the oxidative damage in cisplatin-treated MC3T3-E1 cells was also examined. Firstly, the ROS and superoxide anion were observed using DCFH-DA and Mito-SOX probes, respectively. Figure 5A showed that cisplatin treatment triggered obvious generation of superoxide anion and ROS as imaged by the enhanced fluorescent intensity. Secondly, cisplatin also induced significant DNA damage. For instance, cisplatin (80 μg/ml) significantly activated the phosphorylation of ATR (Ser428), ATM (Ser1981), p53 (Ser15) and histone (Ser139) in a time-dependent manner (Figure 5B). As expected, Se-SP pre-treatment dramatically suppressed cisplatin-induced generation of ROS and superoxide anion, and eventually attenuated cisplatin-induced oxidative damage (Figure 5C). To further investigate the role of ROS, glutathione (GSH), a ROS scavenger was employed. As shown in Figures 5D,E, ROS inhibition by GSH effectively inhibited ROS generation and improved the cell viability in cisplatin-treated cells. Combined treatment of GSH and Se-SP achieved enhanced inhibition of ROS generation and improvement of cell viability, indicating that Se-SP can act as a natural inhibitor of ROS to regulate cisplatin-induced oxidative damage. Taken together, our results revealed that Se-SP had the potential to suppress cisplatin-induced oxidative damage through inhibiting ROS overproduction.

## Discussion

Malignant tumors represent the most common and frequently occurring diseases in the world and show high invasiveness and morbidity (Fokas et al., 2012). Chemotherapy remains one of the major strategies in treating human cancers (Palchaudhuri and Hergenrother, 2007; Chen et al., 2010). Cisplatin-based chemotherapy has achieved great success in clinic in treating kinds of human solid tumors (Palchaudhuri and Hergenrother, 2007; Bruijnincx and Sadler, 2008; Chen et al., 2010). However, the accompanying side effect severely limited its clinical usage, including liver toxicity, cardiotoxicity, nephrotoxicity, neurotoxicity and osteocytotoxicity (Markman, 2003; Takemura and Fujiwara, 2007; Bruijnincx and Sadler, 2008; Soliman et al., 2016). Accumulation of ROS and induction of oxidative damage were accepted as the main anti-malignancy mechanism of cisplatin (Gewirtz, 1999; Minotti et al., 2004). Accumulated evidences have validated that frequent cancer chemotherapy may cause osteoblasts dysfunction and increase the risk of osteoporosis (Cooper, 1997; Hoff and Gagel, 2005). However, little information about cancer chemotherapy-induced osteoblasts dysfunction was available, and the underlying mechanism need to be investigated. In the present study, cisplatin-induced osteoblasts dysfunction was examined in MC3T3-E1 mouse preosteoblast, and the potential protective mechanism of Se-SP was evaluated. The results indicated that Se-SP significantly attenuated cisplatin-induced osteoblasts dysfunction by inhibiting mitochondria-mediated apoptosis and ROS-mediated oxidative damage, which validated that Se-SP was a promising Se species in therapy of oxidative damage-mediated bone diseases.

Apoptosis as a programmed cell death was regulated by variety of genes and proteins factor and showed obvious apoptotic features (Ortiz et al., 2001; Kamachi et al., 2002; Mronga et al., 2004; Orrenius, 2004). Mitochondria is the main regulator in mitochondria-mediated apoptosis pathway, and mitochondrial dysfunction is much related with the lunch of apoptotic signals (Zhu et al., 2016b; Fan et al., 2017b,c). Abnormal cell apoptosis can damage the stability of the cells in the body and promote the production of diseases (Brecht et al., 2005). Oxidative damage was characterized by overproduction of ROS level in tissues or cells, and oxidative damage-mediated abnormal apoptosis significantly increased the potential risk of aging, cancer and diabetes mellitus (Yoo et al., 2009). Recently, evidences showed that oxidative stress was the common pathological mechanism of several types of osteoporosis. It is reported that ROS overproduction-mediated oxidative stress was found in different degrees of postmenopausal osteoporosis, senile osteoporosis and glucocorticoid-induced osteoporosis, indicating that oxidative stress acts key role in the pathological processes of human osteoporosis (Cervellati et al., 2013). Hence, searching novel antioxidants to block oxidative damage-mediated osteoblasts dysfunction has been emerging as potential strategy in chemoprevention and chemotherapy of human osteoporosis. In the present study, cisplatin as a widely used anticancer drug caused significantly cytotoxicity in MC3T3-E1 cells by triggering mitochondria-mediated apoptosis through lunching ROS-mediated oxidative damage. Se-SP pre-treatment significantly alleviated cisplatin-induced preosteoblast toxicity and apoptosis in MC3T3-E1 cells through inhibiting mitochondrial dysfunction and ROS-mediated oxidative damage. Addition of MPTP inhibitor (CsA) and ROS inhibitor (GSH) further confirmed the significant role of mitochondria in cisplatin-induced cells apoptosis of MC3T3-E1 mouse preosteoblast.

Selenium as an essential trace element showed potential in the chemoprevention and chemotherapy of kinds of human diseases (Chen and Wong, 2009a; Yang et al., 2009; Fan et al., 2017b). Chen et al. (2006) reported that *S. platensis* was a good carrier of Se, and the accumulated Se was mainly incorporated into the proteins of *S. platensis* (Brown and Arthur, 2001; Fan et al., 2012). Due to the autofluorescence and its novel pharmacological properties, its protective potential was highly evaluated in recently, including hepatoprotective, antioxidant, radical scavenging, antiarthritic and anti-inflammatory properties (Bashandy et al., 2016; Perez-Juarez et al., 2016). Moreover, studies confirmed that Se incorporation significantly enhanced the antioxidant and anticancer activities of Se-SP (Zhang et al., 2011; Fan et al., 2012; Zhu et al., 2016a). Fan et al. (2012) reported that selenium-containing from selenium-enriched *S. platensis* showed enhanced protective effects against *t*-BOOH-induced oxidative damages and apoptosis in human erythrocytes and hepatocytes by eliminating ROS. In the present study, Se-SP exhibited enhanced ROS inhibition in cisplation-treated cells, and showed enhanced protective effect against cisplatin-induced oxidative damage and apoptosis in MC3T3-E1 cells. Our findings provided evidence that Se-SP could be a promising Se species in chemotherapy and chemoprevention of oxidative damage-mediated bone diseases.

## Ethics Statement

The Study entitled “Selenium-Containing Protein from Selenium-Enriched *Spirulina platensis* Attenuates Cisplatin-Induced Apoptosis in MC3T3-E1 Mouse Preosteoblast by Inhibiting Mitochondrial Dysfunction and ROS-Mediated Oxidative Damage” was performed in Key Lab of Cerebral Microcirculation in Universities of Shandong, Taishan Medical University. All the experiments were performed in accordance with the relevant guidelines and regulations of Taishan Medical University.

## Author Contributions

JO, C-dF, and B-lS designed the experiments. J-yS, X-tF, Y-jH, and J-kM performed the experiments. M-fY and X-yF analyzed the data and images. J-yS and Y-jH wrote the manuscript. All authors reviewed the manuscript.

## Conflict of Interest Statement

The authors declare that the research was conducted in the absence of any commercial or financial relationships that could be construed as a potential conflict of interest.
